# Structures of Wnt-Antagonist ZNRF3 and Its Complex with R-Spondin 1 and Implications for Signaling

**DOI:** 10.1371/journal.pone.0083110

**Published:** 2013-12-12

**Authors:** Weng Chuan Peng, Wim de Lau, Pramod K. Madoori, Federico Forneris, Joke C. M. Granneman, Hans Clevers, Piet Gros

**Affiliations:** 1 Crystal and Structural Chemistry, Bijvoet Center for Biomolecular Research, Department of Chemistry, Faculty of Science, Utrecht University, Utrecht, The Netherlands; 2 Hubrecht Institute, Royal Netherlands Academy of Arts and Sciences and University Medical Centre Utrecht, Utrecht, The Netherlands; Monash University, Australia

## Abstract

Zinc RING finger 3 (ZNRF3) and its homolog RING finger 43 (RNF43) antagonize Wnt signaling in adult stem cells by ubiquitinating Frizzled receptors (FZD), which leads to endocytosis of the Wnt receptor. Conversely, binding of ZNRF3/RNF43 to LGR4-6 – R-spondin blocks Frizzled ubiquitination and enhances Wnt signaling. Here, we present crystal structures of the ZNRF3 ectodomain and its complex with R-spondin 1 (RSPO1). ZNRF3 binds RSPO1 and LGR5-RSPO1 with micromolar affinity via RSPO1 furin-like 1 (Fu1) domain. Anonychia-related mutations in RSPO4 support the importance of the observed interface. The ZNRF3-RSPO1 structure resembles that of LGR5-RSPO1-RNF43, though Fu2 of RSPO1 is variably oriented. The ZNRF3-binding site overlaps with trans-interactions observed in 2:2 LGR5-RSPO1 complexes, thus binding of ZNRF3/RNF43 would disrupt such an arrangement. Sequence conservation suggests a single ligand-binding site on ZNRF3, consistent with the proposed competing binding role of ZNRF3/RNF43 in Wnt signaling.

## Introduction

Zinc RING finger 3 (ZNRF3) and its homolog RING finger 43 (RNF43) are trans-membrane E3 ubiquitin ligases that negatively regulate Wnt signaling [1,2]. Mutations in ZNRF3 or RNF43 have been linked to gastric adenocarcinoma [3], pancreatic ductal adenocarcinoma [4], liver fluke-associated cholangiocarcinoma [5] and mucinous ovarian tumors [6]. ZNRF3 and RNF43 contain an extracellular N-terminal protease-associated (PA) domain, a single pass trans-membrane helix and an intracellular C-terminal RING domain with E3 ligase activity [2]. Interaction of ZNRF3 or RNF43 with complexes of frizzled receptors (FZD) and low-density lipoprotein receptor-related protein (LRP) 5/6 leads to Frizzled ubiquitination and endocytosis of the heterodimeric receptors, thereby reducing the capacity of Wnt-driven signal transduction [1,2]. 

R-spondins 1-4 (RSPO1-4) are stem cell growth factors that bind leucine-rich repeat G-protein coupled receptors 4-6 (LGR4-6) on adult stem cells [7-10], such as in the intestine and colon [11], hair follicles [12], stomach [13], kidney [14], liver [15] and mammary glands [16]. LGR4-6 – R-spondin complexes potentiate Wnt signaling; however, the underlying mechanism is not completely resolved. It was recently reported that LGR4-RSPO1 complex interacts with ZNRF3 and facilitates the removal of ZNRF3 from the membrane, thereby indirectly increasing the number of Wnt receptor/co-receptor complexes on the cell surface [2]. Carmon et al., in contrast, observed that LGR5 forms a supercomplex with FZD-LRP5/6 upon stimulation with R-spondin 1 and Wnt3a and increases the rate of LRP6-FZD receptors internalization and degradation [17]; this model would contradict the role of LGR4/5-RSPO1 in increasing the number of Wnt receptors on the cell surface. 

Recent crystal structures [18-21] showed that RSPO1-4 bind LGR4-6 at the concave surface of the extended leucine-rich repeat (LRR) region of the LGR ectodomain. The ‘phenylalanine clamp’ of RSPO furin-like (Fu) 2 domain is critical for binding to the hydrophobic patch on LRR3-9. In addition, we observed 2:2 LGR5-RSPO1 complexes in four crystal forms [18]. However, such quaternary arrangement was not observed in LGR4-RSPO1 structure [20,21]. 

Here, we present crystal structures of the ectodomain of ZNRF3 and its complex with RSPO1. RSPO1 binds ZNRF3 primarily through its Fu1 domain and Fu2 exhibits domain flexibility in the absence of LGR4/5. Mutations in RSPO4 implicated in congenital anonychia [22] correspond to RSPO1 residues that mediate interactions with ZNRF3. Furthermore, superposition of the ZNRF3-RSPO1 with the LGR5-RSPO1 structures shows that ZNRF3 overlaps with the dimeric partner LGR5 in the 2:2 LGR5-RSPO1 complexes. Thus, interaction of ZNRF3 with LGR5-RSPO1 would block or disrupt this quaternary arrangement.

## Results and Discussion

### Structure of ZNRF3 protease-associated domain

The ectodomain of ZNRF3 was transiently expressed in HEK293 cells. The protein was purified by immobilized metal ion affinity chromatography and gel-filtration. Size-exclusion chromatography and multi-angle laser-light scattering indicated that ZNRF3 exists as monomer in solution (data not shown). Purified protein was crystallized and crystals exhibited space group *P*2_1_ with cell dimensions *a* = 35.7 Å, *b* = 73.5 Å and *c* = 58.6 Å and β = 97.5°, contained two molecules per asymmetric unit and diffracted to 1.5 Å resolution. Crystallographic data and refinement statistics are given in Table 1; electron density is shown in Figure S1A. 

**Table 1:** Crystallographic statistics for data collection and refinement.

	**ZNRF3**	**ZNRF3-RSPO1**
**Data Collection^*a*^**		
X-ray source	SLS X06DA	SLS X06SA
Processing programs	XDS/AIMLESS	iMOSFLM/AIMLESS
Space group	P2_1_	P1
Cell parameters	a = 35.7 Å; α = 90.0°	a = 51.7 Å; α = 66.3°
	b = 73.5 Å; β = 97.5°	b = 80.2 Å; β = 81.4°
	c = 58.6 Å; γ = 90.0°	c = 83.0 Å; γ = 80.7°
Wavelength (Å)	1.00	1.00
Resolution (Å)	45.57 – 1.50 (1.53-1.50)	75.63 – 2.80 (2.95-2.80)
Unique reflections	46197 (4495)	29157 (4105)
CC_1/2_ **^*b*^**	1.00 (0.94)	0.96 (0.21)
Redundancy	2.7 (2.4)	2.2 (2.3)
I/σ(I)	24.1 (4.1)	6.2 (3.2)
Completeness (%)	96.6 (94.0)	95.4 (95.0)
R_sym_^c^	0.019 (0.162)	0.093 (0.391)
Wilson B-factor (Å^2^)	17.66	54.27
**Refinement**		
Molecules per ASU	2	4
R_work_/R_free_^c^	0.162/0.177	0.218/0.246
Average B-factors (Å^2^)	28.1	83.8
Number of atoms:	5184	7982
Protein	4861	7927
Ligands	27	0
Waters	296	55
**Structure quality**		
Molprobity score	1.84	2.05
RMS bond lengths (Å)	0.018	0.005
RMS bond angles (°)	1.83	1.21
Ramachandran favored (%)	98	93
Ramachandran outliers (%)	1	2

^a^ Values in parentheses are for reflections in the highest resolution shell.

_1/2_ (Karplus and Diederichs, 2012).^b^ Resolution limits were determined by applying a cut-off based on the mean intensity correlation coefficient of half-datasets, CC

_sym_ = Σ | I – <I> | / Σ I, where I is the observed intensity for a reflection and <I> is the average intensity obtained from multiple observations of symmetry-related reflections. R_free_ values are calculated based on 5% randomly selected reflections.^c^ R

**Figure 1:**
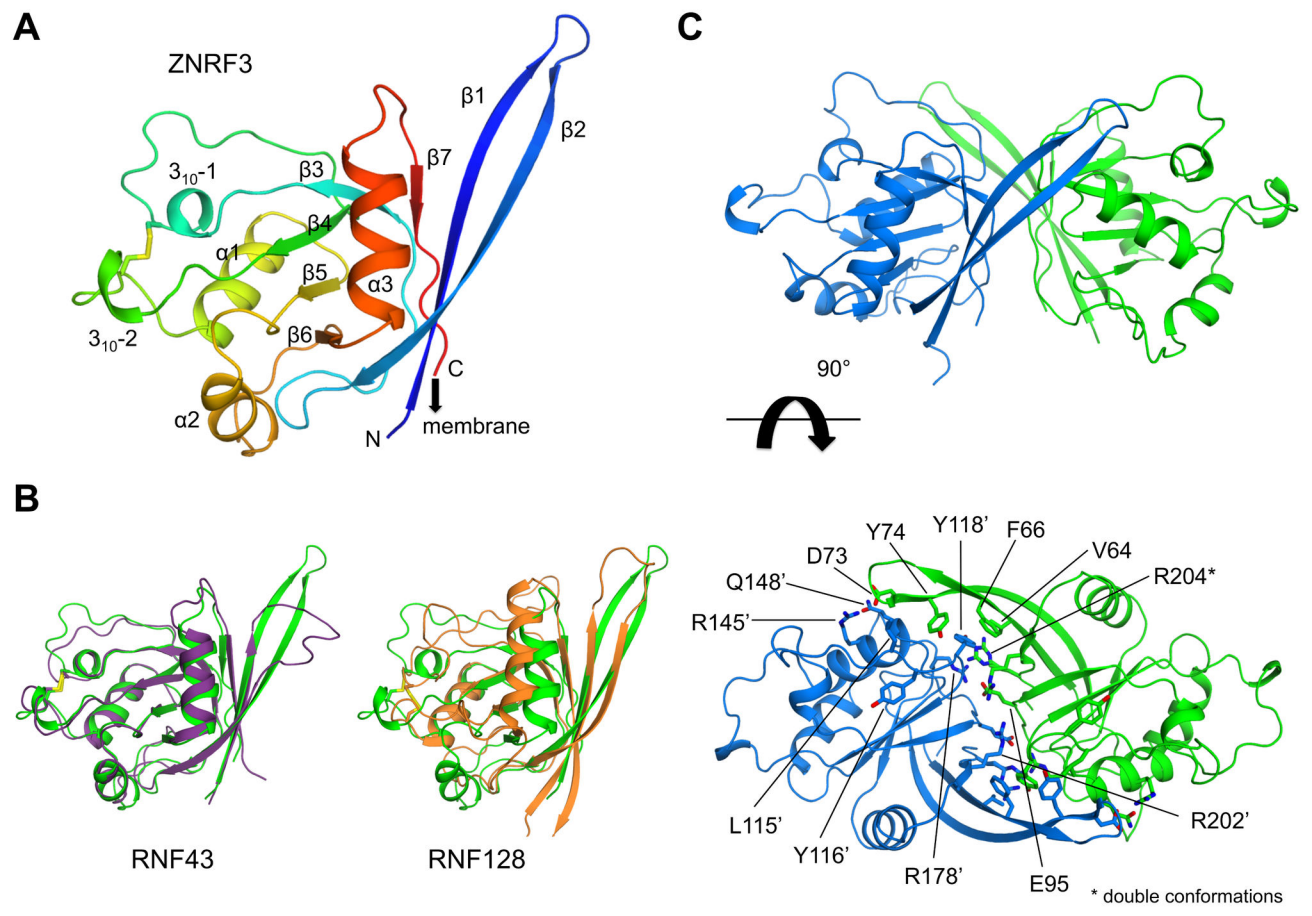
Crystal structure of the ectodomain of ZNRF3. A. Cα trace of ZNRF3 ectodomain coloured from N- to C-terminus (blue through red) and structural elements indicated. The arrow indicates the connection to the single trans-membrane pass. B. Overlay of the Cα trace of ZNRF3 (green) with RNF43 (purple) and RNF128 (orange). C. Two perpendicular views of the dimeric arrangement of ZNRF3 observed in the crystal, with residues at the interface indicated.

The ZNRF3 ectodomain adopts a typical PA-fold, previously found in e.g. subtilases, transferrin receptors and vacuolar sorting receptors [23,24]. The central core of the molecule is formed by a parallel β-sheet, consisting of strands β3-β4-β5-β6 surrounded by three α-helices and two short 3_10_-helices (Figure 1A). A disulphide bond Cys107-Cys136, which is present in both ZNRF3 and RNF43 connects the loop regions containing the first and second 3_10_-helices. The N-terminal and C-terminal residues of the ectodomain form an anti-parallel β-sheet, β2-β1-β7, that packs against helix α3. Consequently, the termini formed by extensions of strands β1 and β7 are close together in space. A linker of approx. 10 residues connects the C-terminus of the ectodomain (Figure 1A) to the trans-membrane helix in the lipid bilayer.

The overall fold of the ZNRF3 ectodomain is similar to the ectodomain of its homolog RNF43, which is in agreement with a sequence identity of 37% between the two ectodomains (Figure 1B and S2). The Cα-positions of ZNRF3 and RNF43 (PDB code 4KNG) [19], can be superimposed on each other with a root-mean-square deviation (rmsd) of 0.75 Å. The largest structural difference between ZNRF3 and RNF43 is observed for the N-terminal strands β1 and β2. In ZNRF3, strands β1 and β2 form an extended β-hairpin ‘flap’, while RNF43 is three residues shorter and displays a flexible loop in this region. Furthermore, DALI search identified RNF128 (also known as GRAIL) to have a related fold (Figure 1B), despite low sequence identity of 15% and a rmsd of 5.3 Å compared to ZNRF3, suggesting that the ectodomains of the Goliath family E3 ligases (such as RNF13, RNF130, RNF133, RNF148, RNF149, RNF150, RNF167 and RNF204) [25] have related folds for ligand recognition.

In the crystal structure of ZNRF3, we observe two molecules in the asymmetric unit (Figure 1C). The two molecules pack together making an extensive interface burying over ~1,000 Å^2^ surface area. The extended β1-β2 flaps fold over the other monomer and provide small hydrophobic and aromatic interaction clusters with Val64, Phe66, Gly72 and Tyr74 interacting with Leu115’ and Y118’ preceding strand β4 and Gly150’ preceding β5 (where prime indicates residues from the opposing dimer), on both sides. Furthermore, a charged and H-bonded network is observed, which includes: Asp73-Arg145’, Tyr74-Tyr116’, Tyr74-Gln148’, Glu95-Glu95’, Glu95-Arg178’ and Glu95-Arg202’. In addition, we observe stacking of guanidinium groups of Arg178-Arg204’. Both C-termini of the dimer point to the same direction, making such arrangement plausible on the membrane. However, at present it is not clear if such arrangement is physiologically relevant.

### Structure of ZNRF3-RSPO1: RSPO1 binds to ZNRF3 through Fu1 domain

We co-crystallized ZNRF3 ectodomain with RSPO1 consisting of the Fu1 and Fu2 domains. RSPO1 was expressed and purified as described previously [18]. Diffraction data were collected up to 2.8 Å resolution from crystals with space group P1 and cell dimension *a* = 51.7 Å, *b* = 80.2 Å, *c* = 83.0 Å and α = 66.3°, β = 81.4°, γ = 80.7° (see Table 1 for crystallographic data and refinement statistics; and Figure S1B for electron density).

**Figure 2:**
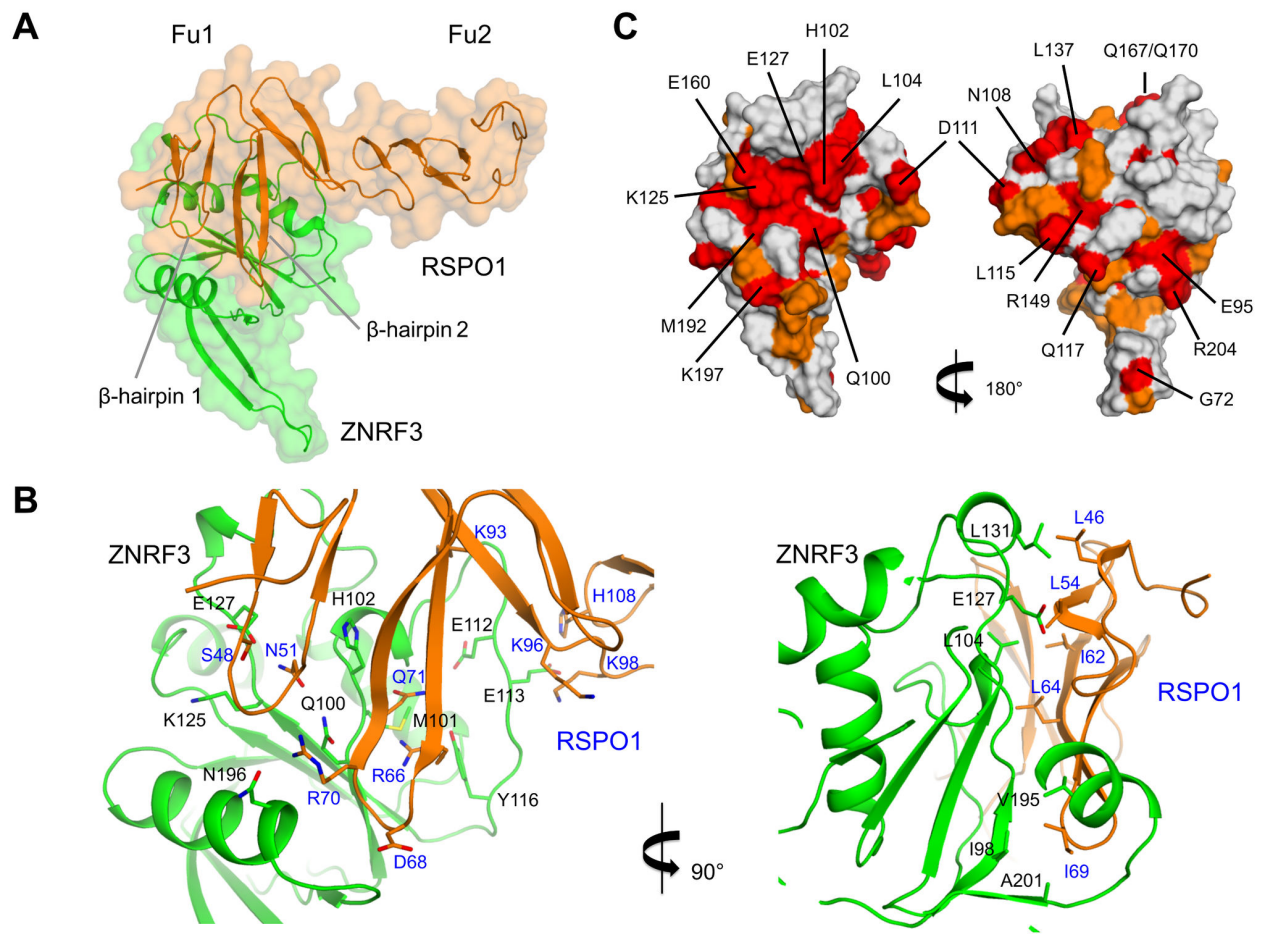
Crystal structure of the ZNRF3-RSPO1 complex. A. Cα trace with transparent surface representation of ZNRF3 (green) and RSPO1 (orange). ZNRF3 makes contacts to the β-hairpins 1-2 of the Fu1 domain of ZNRF3. B. Two views of the binding sites with interface residues indicated. C. Identical (red) and conserved (orange) residues between ZNRF3 and RNF43 are shown in surface representation; two views of ZNRF3 are shown. Identical residues are labeled.

The ZNRF3-RSPO1 complex reveals an extensive interface, burying ~1,200 Å^2^, between the two molecules (Figure 2A,B). ZNRF3 forms a large pocket on the side of the molecule opposite to the C-terminus, which therefore likely faces away from the membrane surface. This binding platform is formed by several structural elements of ZNRF3, involving residues from strand β3, loop β3-β4 containing the first 3_10_-helix, loop β4-α1 and loop α3-β7. Apart from some side-chain rearrangements, no major conformational change is observed in ZNRF3 upon binding to RSPO1.

The interaction site on RSPO1 is formed by the β-hairpins 1 and 2 of the Fu1 domain, which form an extended face that contacts ZNRF3 (Figure 2A). An extensive network of hydrogen bonds and salt bridges mediates the ZNRF3-RSPO1 interaction. The core of the ZNRF3 interface includes residues Gln100, His102, Lys125 and Glu127, which have identical residues in RNF43, as well as residues Met101, Tyr116 and Asn196 that are not conserved in RNF43. These residues interact with the backbones or side chains of residues Ser48, Asn51, Cys53, Arg66, Arg70 and Gln71 of RSPO1 (Figure 2B), which are identical or strongly conserved (Arg70 is Lys in RSPO3) among RSPO1-4. Upon binding to ZNRF3, the tip of β-hairpin 2 (residues ^67^NDIR^70^) becomes ordered, whereas this region was highly flexible in the unbound RSPO1 [18], indicating possible molecular plasticity for binding diverse ligands. The hydrophobic side-chain of Ile69 (with residues Met, Met and Ile at the equivalent position in RSPO2-4, respectively), points into a hydrophobic pocket formed by ZNRF3 and make contacts Ile98 (RNF43: Leu), Val195 (Val) and Ala201 (Ala). Residues Leu46 (RSPO2-4: Ser, Thr, Ile), Leu54 (Ser, Leu, Ser), Ile62 (Phe, Phe, Leu) and Leu64 (Leu, Leu, Ile) lie on one side of β-hairpins 1 and 2 of RSPO1 and their side chains make contacts with Leu104 (RNF43: Leu), Gly105 (Tyr), Glu127 (Glu) and Leu131 (Arg) of ZNRF3 (Figure 2B). However, the residues making hydrophobic interactions are not strictly conserved in other R-spondins (RSPO 2-4). A short stretch of polar and negatively charged residues ^108^,NNNDEED^114^, on ZNRF3 faces residues Lys93, Lys96, Lys98 and His108 on the hinge region of RSPO1. The charge interactions may contribute to long-range attraction between the molecules. However, large B-factor values for ZNRF3 residues located in this region indicate that these contacts are less well defined in the crystal structure.

### A conserved binding platform in ZNRF3/RNF43 and RSPO1-4

The mode of ZNRF3-RSPO1 interaction is consistent with electrostatic interactions observed in RNF43-RSPO1 [19]. The majority of the identical surface-exposed residues cluster on the RSPO1-binding site to form an extended binding platform (Figure 2C and S3A). On the contrary, on the opposite side of the molecule, there are a few scattered identical exposed residues, such as Glu95, Leu115, Gln117 and Arg204 (with neighbouring Arg204 and Glu95 forming a salt bridge); whereas Gly72 and Arg149 expose main-chain atoms only. The presence of one predominant, evolutionary conserved binding platform would indicate that ZNRF3 and RNF43 possibly bind ligands such as Frizzled and RSPO1 at the same or overlapping site. 

On RSPO1, the residues involved in binding ZNRF3, i.e. Ser48, Asn51, Arg66, Arg70 and Gln71, are identical among RSPO1-4 (except for Arg70, which is a lysine in RSPO3). Hence, ZNRF3/RNF43 should be able to bind promiscuously to all R-spondins. 

### Structural flexibility of RSPO1: a hinge region between Fu1 and Fu2 domain

**Figure 3:**
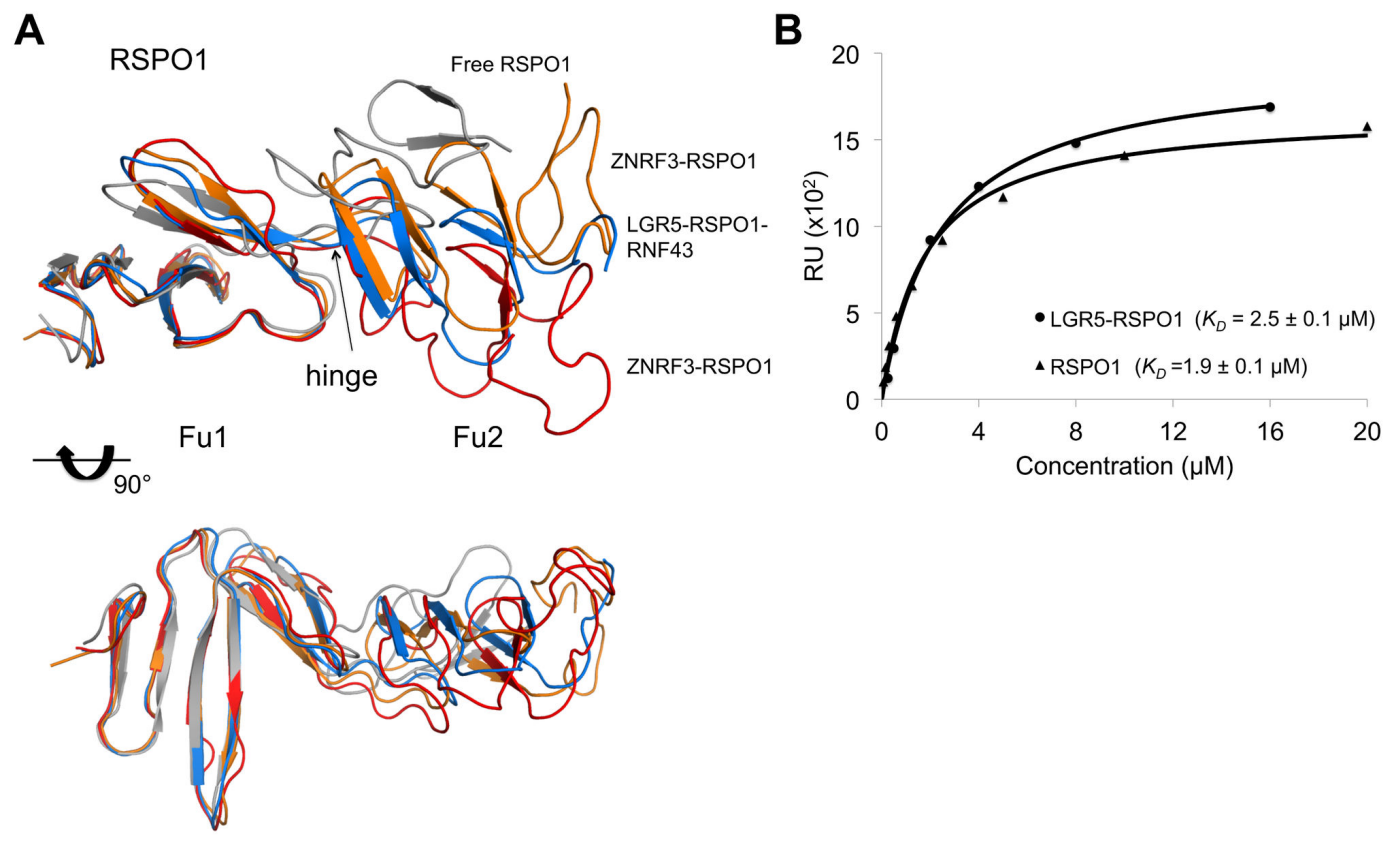
Flexible hinge in RSPO1 and binding of ZNRF3 to RSPO1 and LGR5-RSPO1. A. Overlay of four representative RSPO1 structures in two orientations with free RSPO1 (grey; PDB code 4BSO), RSPO1 in LGR5-RSPO1-RNF43 complex (blue, PDB code 4KNG) and RSPO1 in complex with ZNRF3 (orange and red). B. Representative SPR dose-response curve used to determine equilibrium binding affinity of LGR5-RSPO1 or RSPO1 to ZNRF3, as described in Material and Methods. Standard deviations are calculated from four experiments.

Four copies of ZNRF3-RSPO1 are present in the asymmetric unit (Figure S3B), which are arranged as a dimer of dimers. The dimeric arrangement observed in the structure of ZNRF3 is conserved in the crystal structure of ZNRF3-RSPO1; the two ZNRF3-RSPO1 dimers contact each other sideways through H-bond interactions made by the β1-β2 flaps of ZNRF3. The interactions between ZNRF3 and the Fu1 domain of RSPO1 are identical among the four copies of the complex. Differences, however, are observed with respect to Fu1-Fu2 orientations (Figure 3A). In two copies of RSPO1 (denoted chains F and H) the Fu2 domains are less well packed and display higher B-factors than the other copies (chains E and G; see Figure S3B); these two sets differ by ~20° in Fu1-Fu2 domain orientations. An overlay of RSPO1, RSPO1-ZNRF3 and LGR5-RSPO1-RNF43 structures, reveals a range of Fu1-Fu2 domain orientations with a hinge around residue Lys98. LGR4/5 make interactions with RSPO1 through both Fu1 and Fu2 domains and the LGR4/5-RSPO1 complexes show similar Fu1-Fu2 conformations (PDB codes 4BSR, 4KT1, 4LI2). Binding of RNF43 to LGR5-RSPO1 complex (PDB code 4KNG) does not induce any further conformational change. Apparently, RSPO1 exhibits internal flexibility with a hinge between Fu1 and Fu2; and, this flexibility does not affect ZNRF3 binding, while binding to LGR4/5 straightens the arrangement of the Fu domains.

### Binding studies and the role of LGR5 in interactions with ZNRF3

The RSPO1 and ZNRF3 fragments could not be co-purified by size-exclusion chromatography, indicative of weak binding between RSPO1 and the ZNRF3 ectodomain. To determine whether binding of RSPO1 to ZNRF3 is enhanced by the receptor LGR5, we performed surface-plasmon resonance (SPR) binding studies. LGR5-RSPO1 and RSPO1 bind ZNRF3 with *K*_*D*_ of 2.5 ± 0.1 μM and 1.9 ± 0.1 μM, respectively (Figure 3B). Previously, Chen et al. determined by isothermal titration calorimetry a *K*_*D*_ of 7-10 μM for RSPO1-RNF43 and observed a 10-fold increase in binding affinity (0.5-1.0 μM) in the presence of LGR5 [19]. Superposition of the ZNRF3-RSPO1 and LGR5-RSPO1-RNF43 complexes (Figure S3C) indicates that no contacts are likely between LGR5 and ZNRF3 in LGR5-RSPO1-ZNRF3 either. This observation is consistent with the observed similar binding affinities of RSPO1 and LGR5-RSPO1 to ZNRF3. The structural data do not explain different affinities for binding to RSPO1 and LGR5/RSPO1 as observed for the RNF43. 

Under physiological conditions, LGR5 may function to localize R-spondins on the membrane. Only nanomolar concentrations of R-spondin are required for LGR4-6 binding, Wnt signaling activity and stem-cell driven intestinal organoid growth [7,18]. ZNRF3 functions to ubiquitinate Frizzled receptors, and ZNRF3 itself is targeted by R-spondins for removal from surface [2]. The weak binding affinity observed maybe required for balancing these two events, so that ZNRF3 can exist in equilibrium between RSPO1 and FZD. Previously, Hao et al. detected interaction of ZNRF3 and FZD8 by immuno-precipitation [2]. We tested the binding of ZNRF3 to FZD8 cysteine-rich domain by SPR, the domain responsible for binding to Wnt, but did not observe any binding (data not shown). Hence, ZNRF3 possibly binds to FZD8 outside the cysteine-rich domain or additional factors like Wnt are needed to establish ZNRF3-FZD8 binding. 

### ZNRF3-RSPO1 interface coincides with LGR5-RSPO1 ‘trans’ interfaces

**Figure 4:**
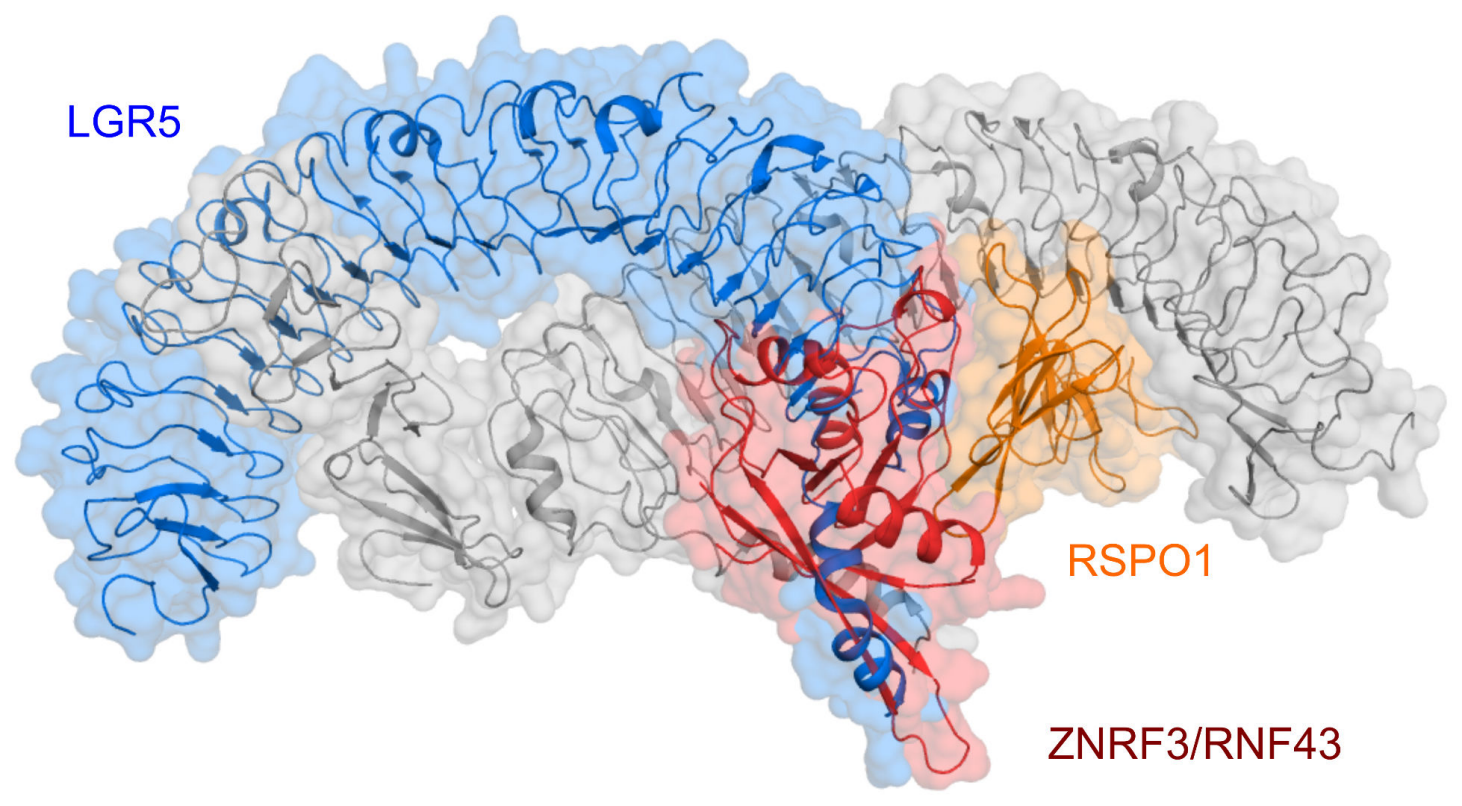
ZNRF3-RSPO1 binding site coincides with LGR5-RSPO1 ‘trans’ site. Shown in cartoon with transparent surface representation are the crystal structure of the 2:2 LGR5-RSPO1 complex (PDB code 4BSR) and the structure of ZNRF3-RSPO1 superimposed on RSPO1 on the right-hand side (orange), only the Cα trace of ZNRF3 (red) is shown for clarity. The overlapping LGR5 chain is shown in blue. The remaining part of the 2:2 LGR5-RSPO1, i.e. left-hand side RSPO1 and right-hand side LGR5, is shown in grey.

Recently, several structures of LGR4/5-RSPO1 complexes were reported [18-21]. These structures were fully consistent with respect to the primary LGR4/5-RSPO1 binding site and interactions, but the structures differed in quaternary arrangements. Whereas Wang et al. and Xu et al. [20,21] observed a 1:1 LGR4-RSPO1 complex, with possible side-to-side contacts between complex in crystal contact, we observed a 2:2 LGR5-RSPO1 complex in multiple crystal forms, where the LRR11-17 repeats are twisted around each other [18]. In the LGR5-RSPO1 dimeric structures, RSPO1 contacts the second copy of LGR5 via the ‘trans’ interface. Specifically, the ‘trans’ interface is formed by Fu1 domain of RSPO1 and C-cap of LGR5. This interface coincides with ZNRF3-RSPO1 interface, also observed in the structure of RNF43 bound to LGR5-RSPO1 (Figure 4). Binding of ZNRF3/RNF43 to LGR5-RSPO1 would therefore disrupt the 2:2 complexes. Indeed in the crystal structure of LGR5-RSPO1-RNF43 an ‘open’ arrangement is observed, which is possibly dimerized sideways stabilized by a Ni^2+^ ion coordinated by residues His199 and His223 from both LGR5 molecules. Of note, Xu et al. observed a LGR4 dimer in solution [21]. Moreover, evaluation of the crystal structure (PDB code 4LI1) shows that LGR4 forms a related dimeric arrangement in the lattice (reminiscent of the reported 2:2 arrangement for LGR5-RSPO1 complexes).

Congenital anonychia is a mild disorder characterized by the absence of fingernails and toenails for which mutations have been identified in RSPO4 [26-29]. These mutations correspond to residues R66W, R70C, Q71R and G73R in RSPO1. To investigate the effect of mutations on Wnt signaling, we have previously performed Wnt reporter assay (TOPFlash) and observed reduced signaling activity [18]. Gln71 and Gly73 residues are located on the ZNRF3-RSPO1 interface and the Anonychia-related mutations, Q71R and G73R, would affect binding to ZNRF3 due to steric clashes and electrostatic repulsion (Figure S3D). Based on the structural data the effects of R66W and R70C are expected to be less severe, because these residues are located at the periphery of the ZNRF3-RSPO1 interface. Indeed, R66W mutant showed slightly higher activity than Q71R and G73R [18], whereas R70C mutant express minimally as monomeric form in HEK 293 cells (data not shown). As described previously, Q71R and G73R may also affect the ‘trans’ LGR5-RSPO1 interactions, whereas R66W and R70C lie outside the observed interface and might be accommodated. Thus, based on the structural data both ZNRF3-RSPO1 and ‘trans’ LGR5-RSPO1 interactions may be affected by Anonychia-related mutations. Moreover, distinguishing between these two types of interactions in functional assays, such as the TOPFlash reporter assay, likely depends critically on the molecular ratio of LGR4-6 and ZNRF3/RNF43 receptors in the membrane. 

## Conclusions

Although a dominant role of LGR4-6 in Wnt activation of adult stem cell maintenance and proliferation have become very clear, the specific contributions of LGR4, LGR5 and LGR6 are not yet fully understood. *Lgr5* expression is specific to stem cell compartments in various tissues, whereas *Lgr4* shows a broader expression pattern [30]. R-spondins have been identified as ligands for LGR4-6, yet signaling does not seem to be coupled to G-proteins [7,9,10]. One recent study reports LGR5 (but not LGR4 or LGR6) activates the G_12/13_-Rho GTPase pathway, but this activity is independent of R-spondins [31]. RNF43 and its homolog ZNRF3 have been identified as E3 ligases [1,2] that ubiquitinate Frizzled receptors for degradation, whereas RSPO1 captures ZNRF3 for removal from membrane, thereby increasing Frizzled expression on the cell surface. Our crystal structures show that ZNRF3 adopts a typical PA domain, which does not undergo major conformational change upon binding to RSPO1. A dimeric arrangement of ZNRF3 is observed, which is plausible on the membrane, though evidence for a physiological role of such a dimer is currently lacking. The structure of ZNRF3-RSPO1 presented here, and the LGR5-RSPO1-RNF43 structure [19], elucidate the mode of interaction between RNF43/ZNRF3 and RSPO1. These structures provide a framework for studying disease mutations, e.g., those in RSPO4 causing congenital Anonychia. RSPO1 binds to ZNRF3 with weak (micromolar) affinity, in contrast to strong (nanomolar) binding affinity for LGR4-6. LGR5 did not increase the affinity of RSPO1 to ZNRF3; this is in contrast to the 10-fold increased affinity for RNF43 reported for LGR5-RSPO1 *versus* RSPO1 alone [19]. Overall, LGR4-6 most likely serve as recruitment receptors providing nanomolar-affinity binding sites for R-spondins on the membrane surface. While the strong affinity allows R-spondin to bind to LGR4-6 at low concentration to become effectively associated to the membrane, the weak affinity for ZNRF3-receptor ectodomain possibly allows for regulation of ubiquitination activity on Frizzled receptors, as proposed by Hao et al. [2]. Moreover, ZNRF3 most likely interacts with R-spondins and Frizzled receptors employing the same conserved binding platform. 

In an earlier report, LGR4 has been found to interact with LGR5 and physically reside in LRP5/6-FZD complexes on the membrane, by tandem affinity purification and mass spectrometry [7]. In another report, Carmon et al. observed that LGR5 forms a supercomplex with LRP6/FZD5 receptors upon stimulation with RSPO1 [17]. Furthermore, LGR5 increased the endocytosis of LRP5/6 complexes in a dynamin- and clathrin-dependent manner. Crystal structures of 2:2 complexes of LGR5-RSPO1 [18] support the observation of LGR4-LGR5 heterodimers. The structures of ZNRF3-RSPO1 and LGR5-RSPO1-RNF43 [19] show that binding of ZNRF3 or RNF43 would disrupt the 2:2 LGR5-RSPO1 complex. These data would indicate the occurrence of multiple types of receptor complexes with potentially different roles, some of which are mutually exclusive. Further, we observed activation of LGR5 by antibodies, in the absence of R-spondin [18], and Kwon et al. showed G_12/13_-Rho GTPase activation of LGR5 independent of R-spondin, implying a direct signaling role apart from that mediated by ZNRF3/RNF43-R-spondin interactions. In addition, RSPO3/4 interacts with Syndecan 4 to activate Wnt/planar cell polarity signaling [8,32]. Other receptors/ligands are also reported to interact with LGR4-6 and/or R-spondins, such as Norrin [33] and Troy [34]. Interestingly, Norrin binds LGR4-6; however, it only activates LGR4. This activation is unlikely mediated by ZNRF3 or RNF43. Various ligands may function to activate different downstream signaling pathway, spatially and temporally, during development. The multitude of proteins involved in Wnt signaling represent an intricate network essential for diverse activity in developmental biology.

## Materials and Methods

### Protein expression, purification and crystallization

LGR5 ectodomain and RSPO1 furin-like domain were expressed and purified as described previously [18]. Mouse ZNRF3 construct (residue 53-205, Uniprot Q5SSZ7; human ZNRF3 residue number was used in the text and structure, i.e., 56-208, Uniprot Q9ULT6) was cloned into pUPE vector (U-Protein Express BV) carrying hexa-histidine tag. All proteins were produced recombinantly in HEK 293E cells that stably expressed Epstein-Barr virus Nuclear Antigen I (EBNA) [35,36] provided by Utrecht-Protein Express BV (Utrecht, The Netherlands). Proteins were purified by Ni-NTA and gel filtration. Samples were concentrated to 10-15 mg/ml in buffer 25 mM HEPES pH 8.0, 50 mM NaCl and crystallized by hanging drop vapour diffusion method at 291 K. Crystals of ZNRF3 were obtained in 0.2M ammonium formate pH 6.6 and 20% w/v PEG 3350. Crystals of ZNRF3-RSPO1 were obtained in 0.2M sodium bromide and 20% w/v PEG 3350. Crystals were harvested and flash-cooled in liquid nitrogen in the presence of mother liquor supplemented with 20% ethylene glycol.

### Data collection, structure determination and refinement

Diffraction data were collected at Swiss Light Source (SLS Villigen, Switzerland) and at European Synchrotron Radiation Facility (ESRF Grenoble, France). Data were processed by MOSFLM [37], XDS [38] and AIMLESS [39]. Resolution limits were determined by applying a cut-off based on the mean intensity correlation coefficient of half-datasets, CC_1/2_ [40]. The structures of ZNRF3 and ZNRF3-RSPO1 were obtained by molecular replacement [41] using RNF43 (PDB code 4KNG) and RSPO1 (PDB code 4BSO) as search models. Model building for ZNRF3 was performed by ARP/WARP [42] and completed manually using COOT [43]. Structure refinements were performed using PHENIX [44] and REFMAC5 [45]. Molprobity [46] was used for structure validation. Structural analysis was performed using various softwares of the CCP4 suite, EBI PISA [47] and the DALI server [48]. Figures were generated with PyMol [49].

### Surface plasmon resonance

Binding studies were performed using IBIS MX96 (IBIS Technologies) according to the protocol described previously [7]. Briefly, ZNRF3 ectodomain or human FZD8 cysteine-rich domain (residue 27-150, Uniprot Q9H461) constructs carrying a C-terminus biotin acceptor peptide (C-BAP) tag were co-expressed with biotin ligase (BirA) in HEK293-E cells to obtain in-vivo biotinylation. Biotinylated ZNRF3 protein was immobilized on a G-streptavidin sensor chip (IBIS Technologies) at different ligand densities. Analytes were flowed on the sensor chip in buffer containing 25 mM HEPES pH 8.0 and 150 mM NaCl at constant temperature of 25 °C. Binding affinities (*K*_*D*_) were calculated by global fitting based on a 1:1 discrete binding mode (SigmaPlot, Systat Software). Standard deviations were calculated from 4 experiments at different ligand density. 

### Accession Numbers

The PDB accession numbers for the coordinates and structure factors of ZNRF3 and ZNRF3-RSPO1 reported in this paper are 4CDJ and 4CDK, respectively.

## Supporting Information

Figure S1 **Electron densities of ZNRF3 and the ZNRF3-RSPO1 complex.** A. Electron density (blue), 2mFo-DFc map contoured at 1 σ level, for ZNRF3. The model is shown in green.
B. Electron density for one of the four ZNRF3-RSPO1 complexes in the asymmetric unit with ZNRF3 in green and RSPO1 in orange. The insert shows a zoom-in of the density at the ZNRF3-RSPO1 interface.
(TIF)

Figure S2 **Sequence alignments of ZNRF3 and RNF43 ectodomains and RSPO1-4.** A. Alignment of human ZNRF3 and RNF43. Mouse ZNRF3 differs from its human homolog at three positions (mouse: His77, Met91 and Leu208).
B. Alignment of human RSPO1-4. The shaded areas correspond to the Fu1-Fu2 domains.
(TIF)

Figure S3 **Structural analyses of ZNRF3-RSPO1 complex.** A. Contact area (‘footprint’) of RSPO1 plotted onto the surface of ZNRF3. ZNRF3 is shown in surface representation with the area in contact with RSPO1 (using a distance criterium of 4.5 Å) highlighted in green. The orientation of the two views is identical as in Figure 2C.
B. Arrangement of the dimer of dimers of ZNRF3-RSPO1 complexes in the asymmetric unit (left side) and the dimeric arrangement based on the ZNRF3 dimer observed in Figure 1C (right side). ZNRF3 molecules are shown in blue and green, RSPO1 in orange and red; the chain labels are indicated.
C. Superposition of the ZNRF3-RSPO1 structure (blue) onto the structure of the LGR5-RSPO1-RNF43 complex (green; PDB code 4KNG).
D. Zoom-in of the ZNRF3-RSPO1 interface, with RSPO1 shown in orange and ZNRF3 in green, highlighting the four residues related to congenital Anonychia mutations in RSPO4: R66W, R70C, Q71R and G73R.
(TIF)
