# Supplementary figures and images for: Structures of Wnt-Antagonist ZNRF3 and Its Complex with R-Spondin 1 and Implications for Signaling
Source: PLoS One. 2013 Dec 12;8(12):e83110. doi: 10.1371/journal.pone.0083110 (PMC3861454; doi:10.1371/journal.pone.0083110)

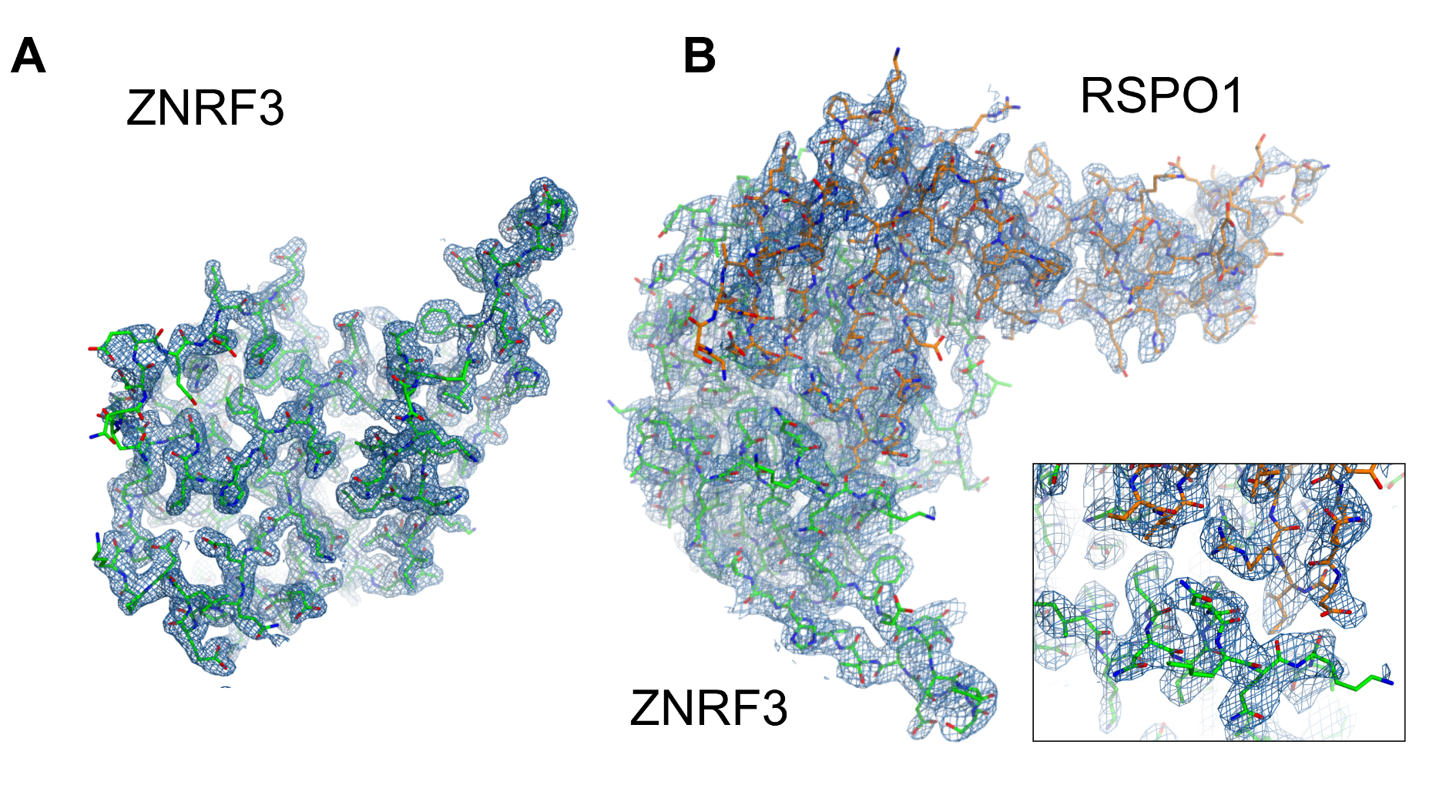

Supplement: Figure S1 — Electron densities of ZNRF3 and the ZNRF3-RSPO1 complex. A. Electron density (blue), 2mFo-DFc map contoured at 1 σ level, for ZNRF3. The model is shown in green. B. Electron density for one of the four ZNRF3-RSPO1 complexes in the asymmetric unit with ZNRF3 in green and RSPO1 in orange. The insert shows a zoom-in of the density at the ZNRF3-RSPO1 interface. (TIF) [file pone.0083110.s001.tif]

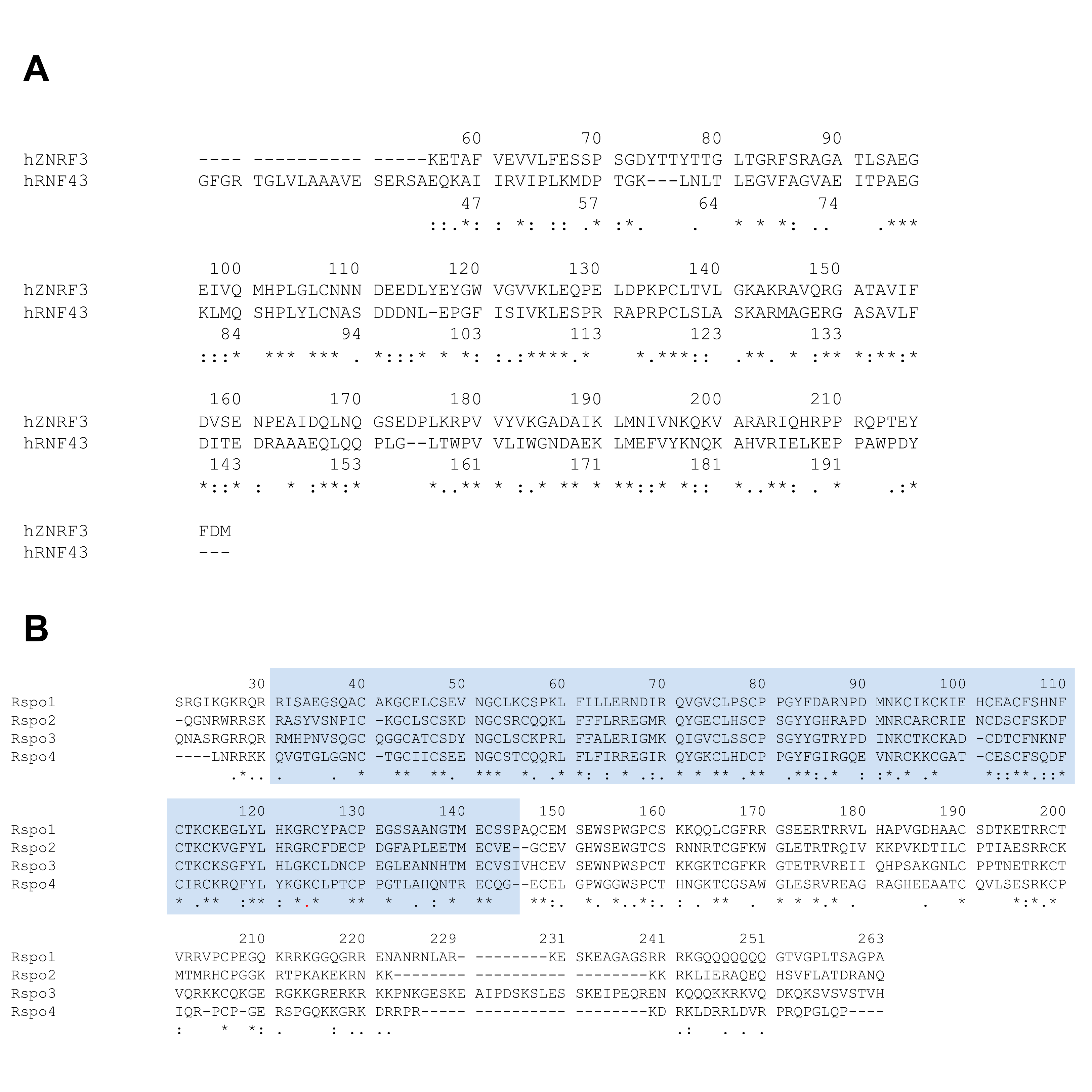

Supplement: Figure S2 — Sequence alignments of ZNRF3 and RNF43 ectodomains and RSPO1-4. A. Alignment of human ZNRF3 and RNF43. Mouse ZNRF3 differs from its human homolog at three positions (mouse: His77, Met91 and Leu208). B. Alignment of human RSPO1-4. The shaded areas correspond to the Fu1-Fu2 domains. (TIF) [file pone.0083110.s002.tif]

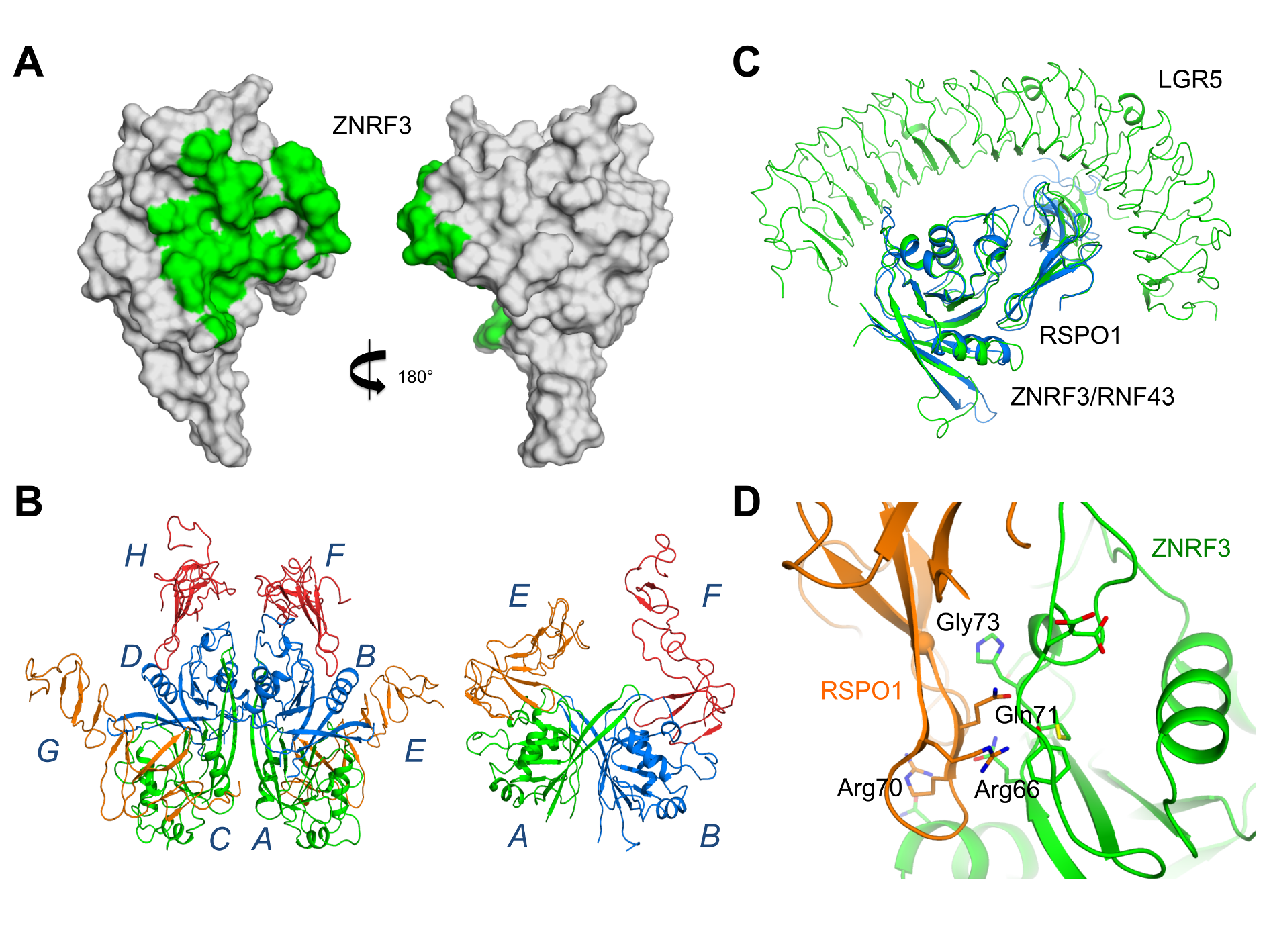

Supplement: Figure S3 — Structural analyses of ZNRF3-RSPO1 complex. A. Contact area (‘footprint’) of RSPO1 plotted onto the surface of ZNRF3. ZNRF3 is shown in surface representation with the area in contact with RSPO1 (using a distance criterium of 4.5 Å) highlighted in green. The orientation of the two views is identical as in Figure 2C. B. Arrangement of the dimer of dimers of ZNRF3-RSPO1 complexes in the asymmetric unit (left side) and the dimeric arrangement based on the ZNRF3 dimer observed in Figure 1C (right side). ZNRF3 molecules are shown in blue and green, RSPO1 in orange and red; the chain labels are indicated. C. Superposition of the ZNRF3-RSPO1 structure (blue) onto the structure of the LGR5-RSPO1-RNF43 complex (green; PDB code 4KNG). D. Zoom-in of the ZNRF3-RSPO1 interface, with RSPO1 shown in orange and ZNRF3 in green, highlighting the four residues related to congenital Anonychia mutations in RSPO4: R66W, R70C, Q71R and G73R. (TIF) [file pone.0083110.s003.tif]
